# Poster Session I - A49 DIRECT TRANSNASAL PLACEMENT OF ENDOLUMINAL VACUUM THERAPY UNDER CONSCIOUS SEDATION: A SINGLE-CENTER CASE SERIES

**DOI:** 10.1093/jcag/gwaf042.049

**Published:** 2026-02-13

**Authors:** N H Phaterpekar, A Dashti, R Trasolini

**Affiliations:** Medicine, The University of British Columbia, Vancouver, BC, Canada; Gastroenterology, University of British Columbia, Vancouver, BC, Canada; Gastroenterology, University of British Columbia, Vancouver, BC, Canada

## Abstract

**Background:**

Endoluminal vacuum (EndoVAC) therapy is a minimally invasive endoscopic approach for managing gastrointestinal (GI) leaks. Conventional systems often require oropharyngeal assembly under general anesthesia, adding airway manipulation, procedural complexity and patient discomfort. We describe a simplified, direct-transnasal EndoVAC technique performed under conscious sedation at a Canadian tertiary centre.

**Aims:**

To evaluate technical and clinical outcomes of a modified EndoVAC technique performed under conscious sedation.

**Methods:**

A retrospective single-centre review was conducted at Vancouver General Hospital including seven patients treated with a modified endoluminal vacuum (EndoVAC) therapy for gastrointestinal (GI) leaks. In this technique, non-adherent Telfa dressings were trimmed to defect size, wrapped around the distal port of a Salem Sump nasogastric tube, and secured with silk suture. The assembly was inserted transnasally under conscious sedation and advanced endoscopically to the defect, where continuous suction was applied. Technical success was defined as successful placement without general anesthesia or intraprocedural complications. Clinical success was defined as complete closure of the leak confirmed endoscopically or radiographically without surgical intervention.

**Results:**

Six patients were treated using repeated exchanges of the modified EndoVAC configuration. The median age was 59 years (range 23–73); four (57%) were male. Indications included three foregut anastomotic leaks, one proximal sleeve leak, and two spontaneous esophageal perforations. Technical success was achieved in all cases (100%). The median number of exchanges was 4 (range 2–6), performed every 3–5 days. The median duration of therapy was 22 days (range 7–28). Clinical success was achieved in five patients (83%), including one who required adjunctive pigtail stenting due to poor tolerance. One patient (17%) required surgical intervention, due to pulmonary communication. Cases were performed under intravenous conscious sedation. There were no serious adverse events.

**Conclusions:**

This simplified, fully transnasal EndoVAC technique eliminates oropharyngeal manipulation and allows atraumatic placement under conscious sedation. It was safe, well tolerated, and achieved outcomes comparable to conventional systems. This approach may broaden the applicability of EndoVAC therapy to patients who are poor anesthetic candidates or in settings with limited access to general anesthesia.

**Funding Agencies:**

None

